# SIRT1 activation with neuroheal is neuroprotective but SIRT2 inhibition with AK7 is detrimental for disconnected motoneurons

**DOI:** 10.1038/s41419-018-0553-6

**Published:** 2018-05-10

**Authors:** David Romeo-Guitart, Tatiana Leiva-Rodríguez, María Espinosa-Alcantud, Núria Sima, Alejandro Vaquero, Helena Domínguez- Martín, Diego Ruano, Caty Casas

**Affiliations:** 1 Institut de Neurociències (INc) and Department of Cell Biology, Physiology and Immunology, Centro de Investigación Biomédica en Red sobre Enfermedades Neurodegenerativas (CIBERNED), Universitat Autònoma de Barcelona (UAB), Barcelona, Spain; 2 Chromatin Biology Laboratory, Cancer Epigenetics and Biology Program (PEBC), Bellvitge Biomedical Research Institute (IDIBELL), L´Hospitalet de Llobregat, Barcelona, Spain; 3 Departamento de Bioquímica y Biología Molecular, Facultad de Farmacia, Instituto de Biomedicina de Sevilla (IBiS), Hospital Universitario Virgen del Rocío/Consejo Superior de Investigaciones Científicas, Universidad de Sevilla, Sevilla, 41012 Spain

## Abstract

Sirtuin 1 (SIRT1) activity is neuroprotective, and we have recently demonstrated its role in the retrograde degenerative process in motoneurons (MNs) in the spinal cord of rats after peripheral nerve root avulsion (RA) injury. SIRT2 has been suggested to exert effects opposite those of SIRT1; however, its roles in neurodegeneration and neuron response after nerve injury remain unclear. Here we compared the neuroprotective potentials of SIRT1 activation and SIRT2 inhibition in a mouse model of hypoglossal nerve axotomy. This injury induced a reduction of around half MN population within the hypoglossal nucleus by a non-apoptotic neurodegenerative process triggered by endoplasmic reticulum (ER) stress that resulted in activation of the unfolded protein response mediated by IRE1α and XBP1 by 21 days post injury. Both SIRT1 activation with NeuroHeal and SIRT2 inhibition with AK7 protected NSC-34 motor neuron-like cells against ER stress in vitro. In agreement with the in vitro results, NeuroHeal treatment or SIRT1 overexpression was neuroprotective of axotomized hypoglossal MNs in a transgenic mouse model. In contrast, AK7 treatment or *SIRT2* genetic depletion in mice inhibited damaged MN survival. To resolve the in vitro/in vivo discrepancies, we used an organotypic spinal cord culture system that preserves glial cells. In this system, AK7 treatment of ER-stressed organotypic cultures was detrimental for MNs and increased microglial nuclear factor-κB and the consequent transcription of cytotoxic pro-inflammatory factors similarly. The results highlight the importance of glial cells in determining the neuroprotective impact of any treatment.

## Introduction

Axonopathy is a common early characteristic of the neurodegenerative processes in the central nervous system^1^. Axon degeneration often leads to retrograde neuronal cell death or atrophy and the progressive and permanent loss of vital neuronal functions. Deciphering the signaling involved is necessary for the development of effective neuroprotectants that are greatly needed in the clinics. Transgenic models of neurodegenerative diseases are broadly used, but shed light on only single pieces of the puzzle. Non-transgenic models of neurodegeneration also yield valuable information regarding the “naturally occurring processes” after neuronal soma–axon disconnection. By exploiting the anatomical and technical advantages of several axotomized models, it has been shown that multiple signaling programs operate in parallel in neuronal soma during the retrograde neurodegenerative process that does not end in apoptosis^2, 3^.

This knowledge has been useful in the discovery of effective neuroprotectants such as the new discovered drug combination NeuroHeal (NH)^4, 5^. NH was discovered using unbiased proteomic data, systems biology, and artificial neural networks from two models that represented pure regenerative and pure neurodegenerative conditions after peripheral nerve distal axotomy or root avulsion (RA) injuries, respectively. The data were used to build up bona fide state-specific molecular maps, and mathematical models were used to screen databases of drugs to identify putatively neuroprotective combinations. NH is a combination of acamprosate and ribavirin, two repurposed drugs with well-described pharmacokinetics and pharmacodynamics profiles that have no serious adverse effects.

Activation of sirtuin 1 (SIRT1) was shown to be necessary for the neuroprotective action of NH after RA^5^. Sirtuins constitute a highly conserved family of deacetylases that regulate cellular homeostasis^6^. Seven homologs of yeast Sir2 (SIRT1–7) that have a conserved catalytic domain have been identified in mammals^7^. Among them, SIRT1 and SIRT2 have been targeted in the design of neuroprotectants for the treatment of degenerative conditions and traumatic injuries^8^. SIRT1 regulates a number of pathways associated with normal metabolism and functioning of individual organs in mammals^9^, including those involving histone 3, p53, and nuclear factor-κB (NF-κB) among others^10^. SIRT2 regulates cell cycle and genome instability in part acting as a histone deacetylase with a preference for histone H4 lysine 16^11^. It is also the most abundant sirtuin in the brain where it deacetylates α-tubulin at lysine 40 contributing in microtubule dynamic regulation^12^.

Both SIRT1 activation and SIRT2 inhibition have been identified as good strategies for neurodegenerative diseases. In the present work, we aimed to validate whether these strategies could be extended to disconnected neurons using a model of axotomy of the hypoglossal nuclei, which contain highly vulnerable cranial MNs^13–15^ that differ in their properties from the spinal MNs^16^.

## Results

**Fig. 1:**
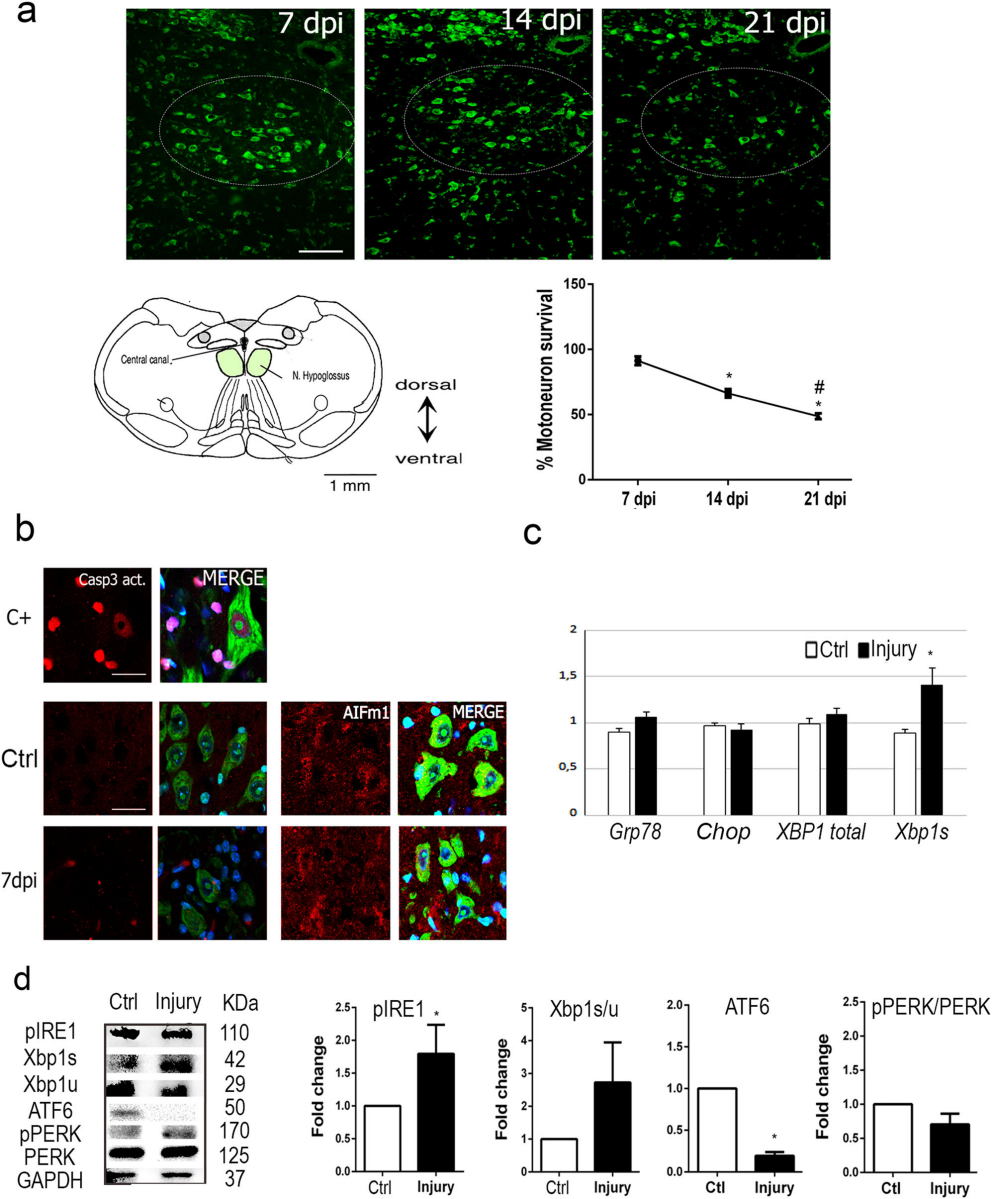
Hypoglossal nerve injury triggers non-apoptotic MN death with activation of IRE1α and XBP1. **a** (Top) Microphotographs of Fluoro-Nissl-stained MNs from the ipsilateral hypoglossal nucleus (dotted circled) from HA-injured animals at 7, 14, and 21 dpi. (Bottom left) Drawing highlighting the hypoglossal nuclei in the brainstem of the mice. (Bottom right) Graphic showing the average percentages ± SEMs of MN survival at the ipsilateral side with respect to the contralateral after HA at indicated dpi (*n* = 4 for 14 dpi and *n* = 5 for other groups; analysis of variances (ANOVA), post hoc Bonferroni; ^#^*p* < 0.05 vs. 7 dpi and *p < 0.05 vs. 14 dpi). **b** (Top panels) Representative images of positive control (C+) from spinal cord samples of neonatal rats submitted to sciatic crush injury stained for active cleaved caspase-3 (Casp3.Act) merged with Fluoro-Nissl (green) and DAPI (blue). (Middle and bottom panels) Representative confocal images of MNs from hypoglossal nuclei immunolabeled for Casp3.Act or AIF1m (red) merged with Fluoro-Jade C-stained hypoglossal nuclei (green) counterstained with DAPI from control (Ctrl) non-injured mice or HA-injured mice at 7 dpi. Scale bars = 25 μm. **c** Bar graphs of the expression levels of different UPR-target genes. **d** Western blots and bar graphs showing the analysis of phosphorylated IRE1α and PERK, the cleaved fragment of activated ATF6, and spliced (Xbp1s) and unspliced (Xbp1u) XBP1 protein levels in Ctrl and HA-injured animals at 3 dpi

In order to evaluate how SIRT1 and SIRT2 function in neuroprotection of MNs after proximal nerve injury we chose to use the hypoglossal axotomy model^17^ (Fig. 1a). We first characterized the pace of MN cell death at the hypoglossal nucleus over time post injury. We observed that the process slowly proceeded with the number of MNs reduced by 48.72 ± 2.2% after 21 days post injury (dpi) (Fig. 1a). Apoptotic hallmarks, such as activation of caspase-3 or apoptosis-inducing factor (AIF) nuclearization, were not observed, although MNs were degenerating as revealed by Fluoro-Jade C staining at 7 dpi (Fig. 1b). These observations were similar to those in our previously reported model of nerve RA in rat^18^. To explore whether there was activation of endoplasmic reticulum (ER) stress in the hypoglossal axotomy model, we analyzed the expression of target genes of the unfolded protein response (UPR) activated after ER stress, as *Chop*, *Grp78*, *Xbp1*, and its spliced version (*Xbp1s*), as well as the activation of three main branches of UPR, PKR-like ER kinase (PERK), inositol-requiring enzyme 1α (IRE1α), and activating transcription factor 6 (ATF6), by western blot. The only gene found overexpressed was the spliced form of *Xbp1* at 3 dpi (Fig. 1c). The apparition of this form after ER stress depends on the activation of IRE1α, which autophosphorylates, dimerizes, and acquires ribonuclease activity, leading to splicing of the mRNA encoding the X-box binding protein XBP1^19^. In agreement, we observed that the phosphorylated dimer of IRE1α was more abundant and the ratio of spliced to unspliced XBP1 was higher in animals subjected to hypoglossal axotomy than controls by western blot (Fig. 1d). However, there were not activation of the UPR branches ATF6 or PERK after 3 dpi characterized by the presence of the 50-kDa-cleaved fragment of ATF6 and the increase of the phosphorylated isoform of PERK, respectively (Fig. 1d). These results suggest that the neurodegenerative pathway involved in cranial MN death is non-apoptotic and presents some hallmarks for the presence of ER stress, similar to the process that occurs after RA in rats were only IRE1α activation was also found.

**Fig. 2:**
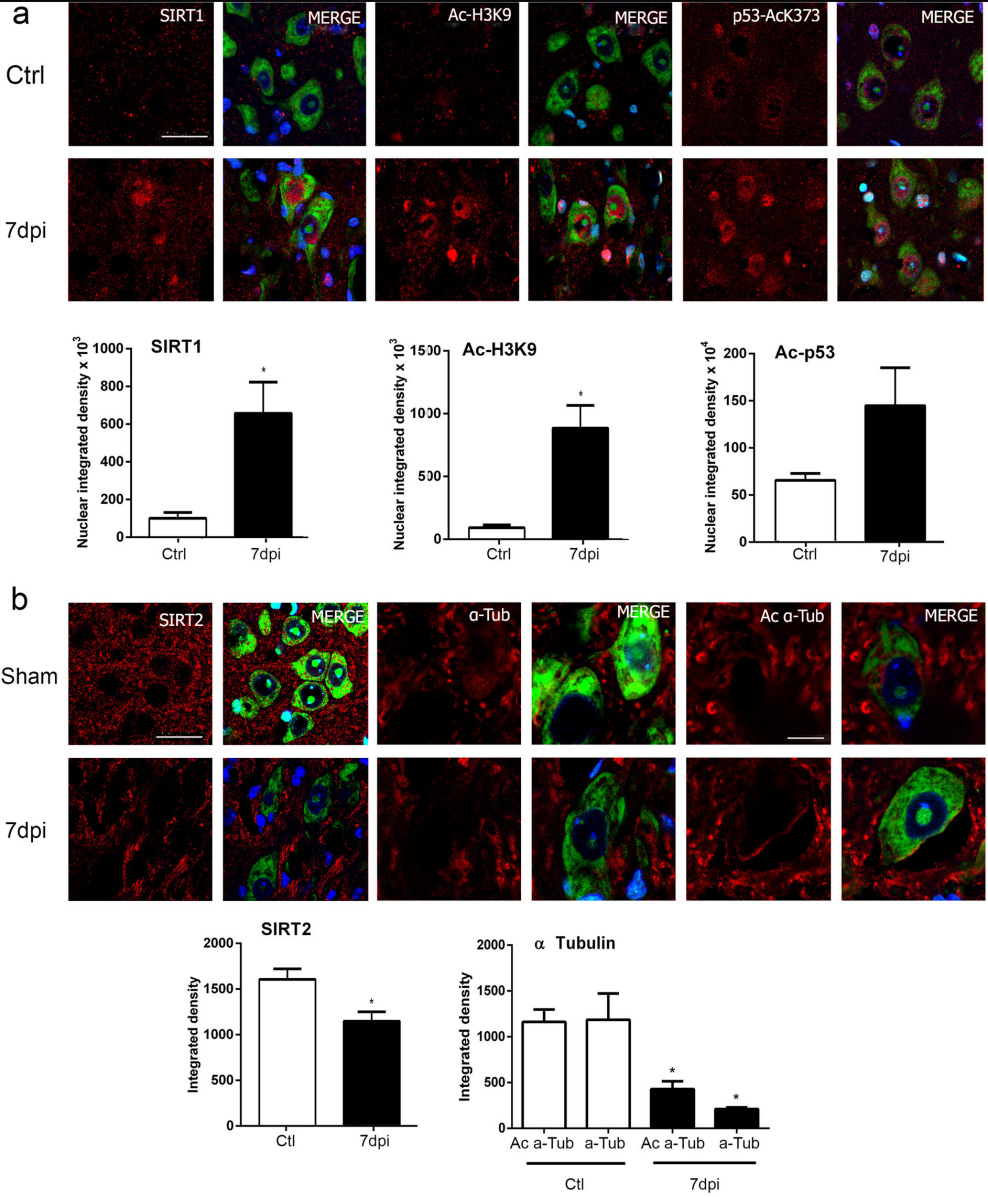
Deacetylase activities of SIRT1 and SIRT2 are reduced after nerve injury. **a** (Top) Confocal images of MNs immunolabeled for SIRT1, Ac-H3K9, and Ac-p53K373 (red) and counterstained with Fluoro-Nissl (green) and DAPI (blue) at hypoglossal nuclei of non-injured control (Ctrl) and HA-injured animals at 7 dpi. Scale bar = 25 µm. (Bottom) Histograms of the means of immunofluorescence densities for each marker inside nuclei of MNs (*n* = 4 animals, *t* test, *p < 0.05 vs. Ctrl). **b** (Top) Representative confocal microphotographs of SIRT2, α-tubulin (α-Tub), and acetylated α-tubulin (Ac α-Tub) in red, counterstained with green Fluoro-Nissl and DAPI from hypoglossal nuclei of non-injured or injured animals at 7 dpi. Scale bar = 25 µm. (Bottom) Histograms of the means of the immunofluorescence densities for each marker inside nuclei of MNs (*n* = 4 animals, *t* test, *p < 0.05 vs. Ctrl). Scale bar = 25 µm and 10 µm for α-Tub and acetyl α-Tub staining

Activation of SIRT1 or inhibition of SIRT2 promotes neuroprotection in some neurodegenerative mouse models^20^. Thus, we analyzed the effect of axotomy of the hypoglossal nerve on the expression and activity of SIRT1 and SIRT2. SIRT1 was overexpressed in the nuclei of axotomized MNs at 7 dpi (Fig. 2a). As a measure of its deacetylase activity, we evaluated levels of histone 3, which is acetylated at lysine 9 (Ac -H3K9), and p53, which is deacetylated at lysine 373 (Ac-p53K373), by SIRT1 as previously reported^21, 22^. Both acetylated substrates significantly accumulated in the nuclei of damaged MNs at 7 dpi when compared to sham-operated control animals (Fig. 2a). SIRT2 appeared at a characteristic location probably corresponding to the ER–Golgi intermediate compartment^23^, and it was present at lower levels in axotomized MNs at 7 dpi with respect to the control (Fig. 2b). Although the total abundance of α-tubulin was diminished after injury, relative levels of the acetylated form of α-tubulin were higher than in controls (Fig. 2b). These results suggest that activities of both SIRT1 and SIRT2 are reduced early after injury.

**Fig. 3:**
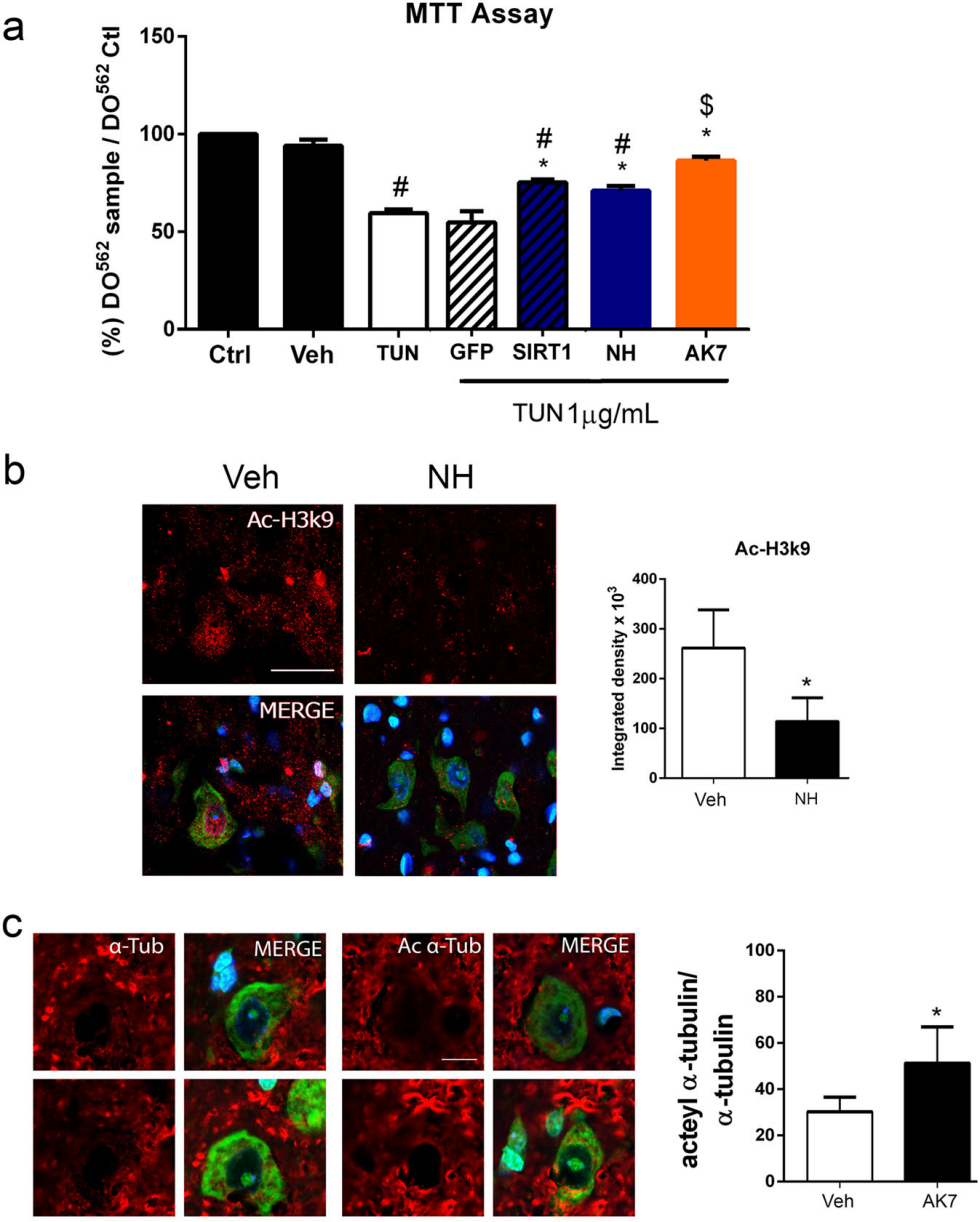
SIRT1 activation by NH and SIRT2 inhibition by AK7 are neuroprotective in ER-stressed NSC-34 MN-like cell culture. **a** Bar graph showing the percentages of cells ± SEMs that survived after the treatment with vehicle (Veh) 1 μg/ml tunicamycin (TUN) alone or in combination with the NeuroHeal or AK7. Percent cell survival was also determined for CMV-SIRT1 and CMV-GFP cells treated with TUN (*n* = 4–10, analysis of variances (ANOVA), post hoc Bonferroni ^#^*p* < 0.05 vs. Veh, *p < 0.05 vs. TUN, ^$^*p* < 0.05 vs. TUN-GFP). **b** (Left) Confocal images of H3K9ac (red) in samples from mice subjected to hypoglossal axotomy and treated with vehicle or NH, counterstained with Fluoro-Nissl (green) and DAPI (blue) at 21 dpi. Scale bar = 25 µm. (Right) Histogram showing the amount of fluorescent density of nuclear H3K9ac in injured MNs in vehicle or NH-treated groups at 21 dpi (*n* = 4, *t* test, *p < 0.05 vs. Veh). **c** (Left) Microphotographs showing acetylated and total α-tubulin (red) staining with Fluoro-Nissl (green) and DAPI (blue) counterstaining in animals treated with vehicle or AK7 at 21 dpi. Scale bar = 10 µm. (Right) Histogram showing the ratio of the acetylated vs. total α-tubulin within the MNs in both groups (*n* = 4, *t* test, *p < 0.05 vs. Veh)

We used an in vitro model of ER stress to test whether an SIRT1 activation and SIRT2 inhibition were neuroprotective. We used tunicamycin (TUN) as stressor agent on NSC-34 cells, which are MN like. After 24 h, TUN treatment had induced death of 59.58 ± 1.82% of cells (Fig. 3a). Overexpression of SIRT1 promoted survival of ER-stressed cells compared to GFP-overexpressing insulted cells with only about 20% of cells dead after 24 h of TUN treatment (Fig. 3a). Treatment of ER-stressed cells with NH, which activates SIRT1 (Fig. S1), was also protective (Fig. 3a). To analyze effects of SIRT2, we used AK7, a SIRT2 inhibitor previously reported to exert neuroprotection in some models of neurodegenerative diseases. Treatment of the stressed cells with AK7 was also neuroprotective. We monitored AK7 activity by analyzing the levels of acetylated H4 at lysine 16 (Ac-H4K16) (Fig. S2).

We then evaluated effects of administration of these drugs in vivo. Oral NH treatment in the hypoglossal axotomy model led to a decrease of Ac-H3K9 levels within the nucleus of MNs compared to vehicle treatment (Fig. 3b). Similarly, intraperitoneal (i.p.) AK7 treatment increased acetylated α-tubulin levels in the hypoglossal nuclei at 7 dpi with respect to control, as expected for a SIRT2 inhibitor (Fig. 3c).

**Fig. 4:**
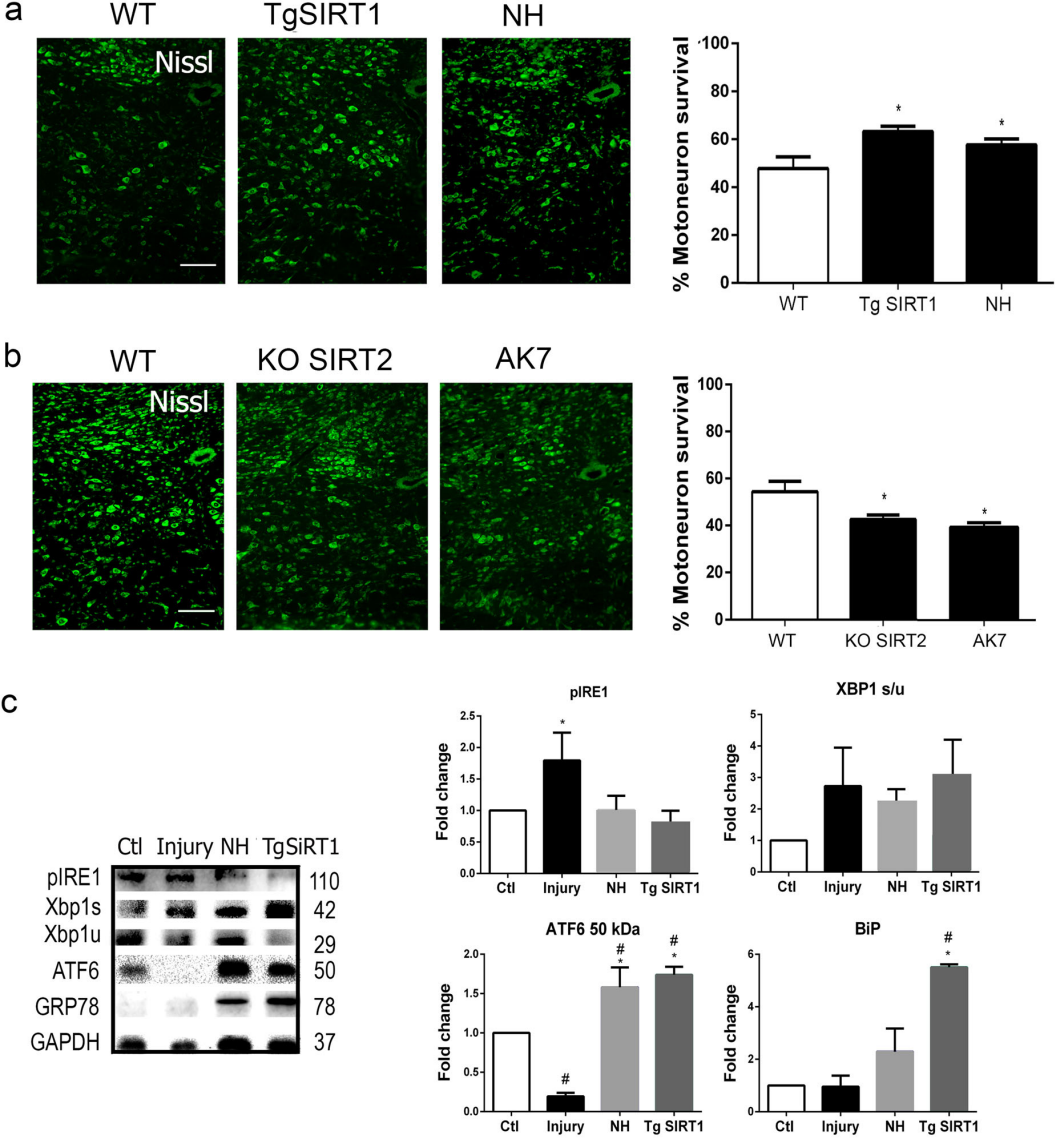
SIRT1 and SIRT2 activities are essential for survival of MNs after HA. **a** (Left) Representative microphotographs of Fluoro-Nissl-stained ipsilateral hypoglossal nuclei MNs from injured WT mice, tg-SIRT1 mice, or NH-treated WT mice at 21 dpi. Scale bar = 100 µm. (Right) The histogram represents the mean percentages ± SEMs of surviving MNs in the ipsilateral side respect to the contralateral side (*n* = 4–5, analysis of variances (ANOVA), post hoc Bonferroni, *p < 0.05 vs. WT). **b** (Left) Hypoglossal nuclei stained with Fluoro-Nissl from WT mice, SIRT2-KO mice, and AK7-treated WT mice (20 mg/kg i.p, daily) at 21 dpi. Scale bar = 100 µm. (Right) Plots of the percentages ± SEMs of the counted MNs at the ipsilateral side respect the contralateral for different experimental groups at 21 dpi (*n* = 4, ANOVA, post hoc Bonferroni, *p < 0.05 vs. WT). **c** Western blots and bar graphs showing the analysis of phosphorylated IRE1α, the cleaved fragment of activated ATF6, spliced (Xbp1s) and unspliced (Xbp1u) XBP1 and GRP78 protein levels in Ctrl, HA-injured, and NH-treated HA-injured wild-type animals and HA-injured tg-SIRT1 animals at 3 dpi

These results suggest that NH and AK7 are neuroprotective; we used both pharmacological and genetic approaches to test these hypotheses. Injured animals treated with NH had an increased percentage of MN survival than vehicle-treated injured mice (Fig. 4a). Similarly, a higher percentage of MN survival was observed post injury in transgenic animals overexpressing SIRT1 (tg-SIRT1)^24^ compared to injured wild-type (WT) littermates (Fig. 4a). We also compared survival of MNs in injured mice that lack SIRT2 (*SIRT2* knockout or KO-SIRT2 mice)^25^ compared to WT littermates with and without treatment with vehicle or AK7 at 21 dpi (Fig. 4b). Unexpectedly, we observed a reduction in the number of surviving MNs in injured KO-SIRT2 mice relative to WT controls and in injured KO-SIRT2 mice treated with AK7 compared to vehicle-treated mice. Since, SIRT1 activation or overexpression resulted neuroprotective, we explored the effect on IRE1α-neuroprotective and ATF6-neuroprotective UPR branches and found a marked increase in cleaved ATF6 and its downstream target GRP78 (Fig. 4c). No significant modifications were observed regarding IRE1α activation and downstream XBP1 splicing. These results suggest that inhibition of SIRT2 is detrimental in vivo, although not in vitro, whereas fostering SIRT1 activity is beneficial for MN survival after axotomy of the hypoglossal nerve. Furthermore, SIRT1 activation or overexpression reinforce endogenous mechanisms of neuroprotection related to UPR.

**Fig. 5:**
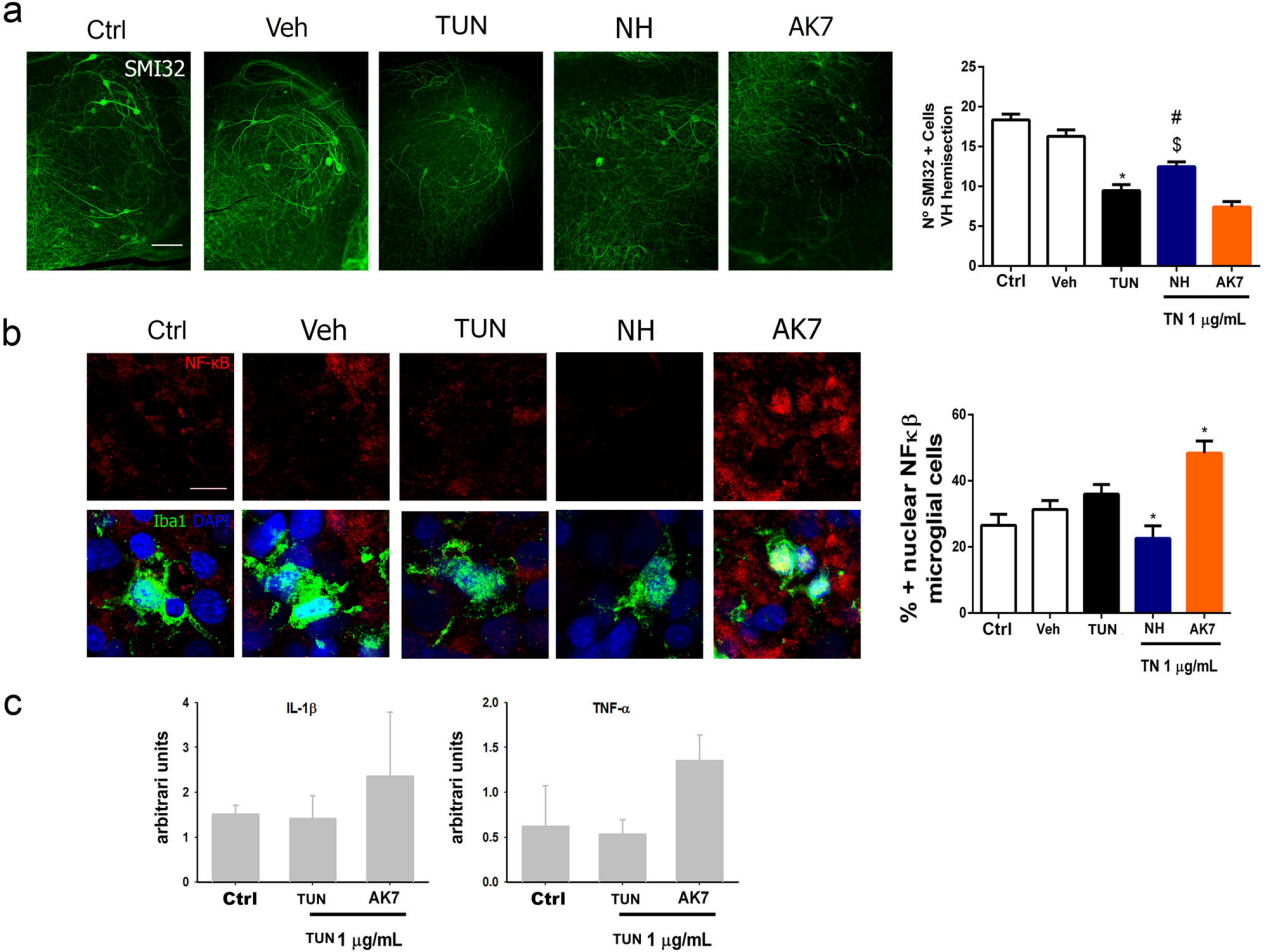
SIRT1 and SIRT2 activities maintain neuron survival in an in vitro model of ER stress in SCOCs. **a** (Left) Representative microphotographs of ventral horn of the SCOCs stained for SMI-32 after 2 days of treatment with TUN with or without the NH or AK7 co-treatment. Scale bar = 100 µm. (Right) Bar graphs showing the numbers ± SEMs of SMI-32-positive cells in the ventral horns of each hemisection of the spinal cord slice (*n* = 5–10 complete spinal cord slices, analysis of variances (ANOVA), post hoc Bonferroni *p < 0.05 vs. Veh, ^#^*p* < 0.05 vs. TUN, ^$^*p* < 0.05 vs. AK7). **b** (Left) Confocal microphotographs stained for the p65 subunit of NF-κB (red) and Iba1, a microglia marker (green). Samples were counterstained with DAPI (blue). Samples from different experimental conditions were stained 6 h after TUN addition. Scale bar = 10 µm. (Right) Histogram showing the percentages of microglial cells with positive nuclear labeling of NF-κB for each experimental group (*n* = 4, ANOVA post hoc Dunnett's test, *p < 0.05 vs. TUN). **c** Histogram of mean values obtained by quantitative real-time PCR for *IL-1β* and *TNFα* mRNA in the control (Ctrl), TUN, or TUN plus AK7 SCOCs at 6 h post treatment

Due to differences between in vitro and in vivo results regarding neuroprotection provided by AK7 treatment, we evaluated this drug in a spinal cord organotypic culture (SCOC) model^26^ as we speculated that lack of glial cells in the culture might be the cause of this discrepancy. In the SCOC, TUN treatment produced a decline in SMI-32-positive MN cells compared to untreated or vehicle-treated SCOCs (Fig. 5a). We observed that TUN treatment drastically reduced the activity of SIRT1 as was the case for nerve injury in vivo, but in the SCOC, this stress enhanced the activity of SIRT2 (Figs. S1 and 2). NH treatment of TUN-treated SCOCs enhanced survival of MNs, whereas AK7 treatment yielded similar results to TUN treatment alone (Fig. 5a). Hence, for AK7, the results obtained in SCOCs were unlike those obtained in the NSC-34 model but similar to those obtained in the in vivo model.

Considering that one of the main differences between the in vitro models is whether or not glia cells are present, we hypothesized that AK7 influences this particular cell type to alter MN destiny. One of the possibilities to be explored was that AK7 treatment renders microglia more reactive^27^. As the transcription factor NF-κB is involved in the production of pro-inflammatory cytokines^27^, we investigated its protein levels in the nuclei of microglia in SCOCs. We did not observe a significant increase of NF-κB in TUN-treated SCOCs compared to levels in vehicle-treated or control cultures (Fig. 5b). In contrast, there was a considerable decrease in nuclear NF-κB in the microglia of NH-treated stressed SCOCs, whereas AK7 treatment significantly increased NF-κB levels in these cultures (Fig. 5b). Accordingly, levels of interleukin-1β (*IL-1β*) and tumor necrosis factor-α (*TNFα*) expression were increased in AK7-treated SCOCs with respect to TUN-treated or control samples (Fig. 5c).

**Fig. 6:**
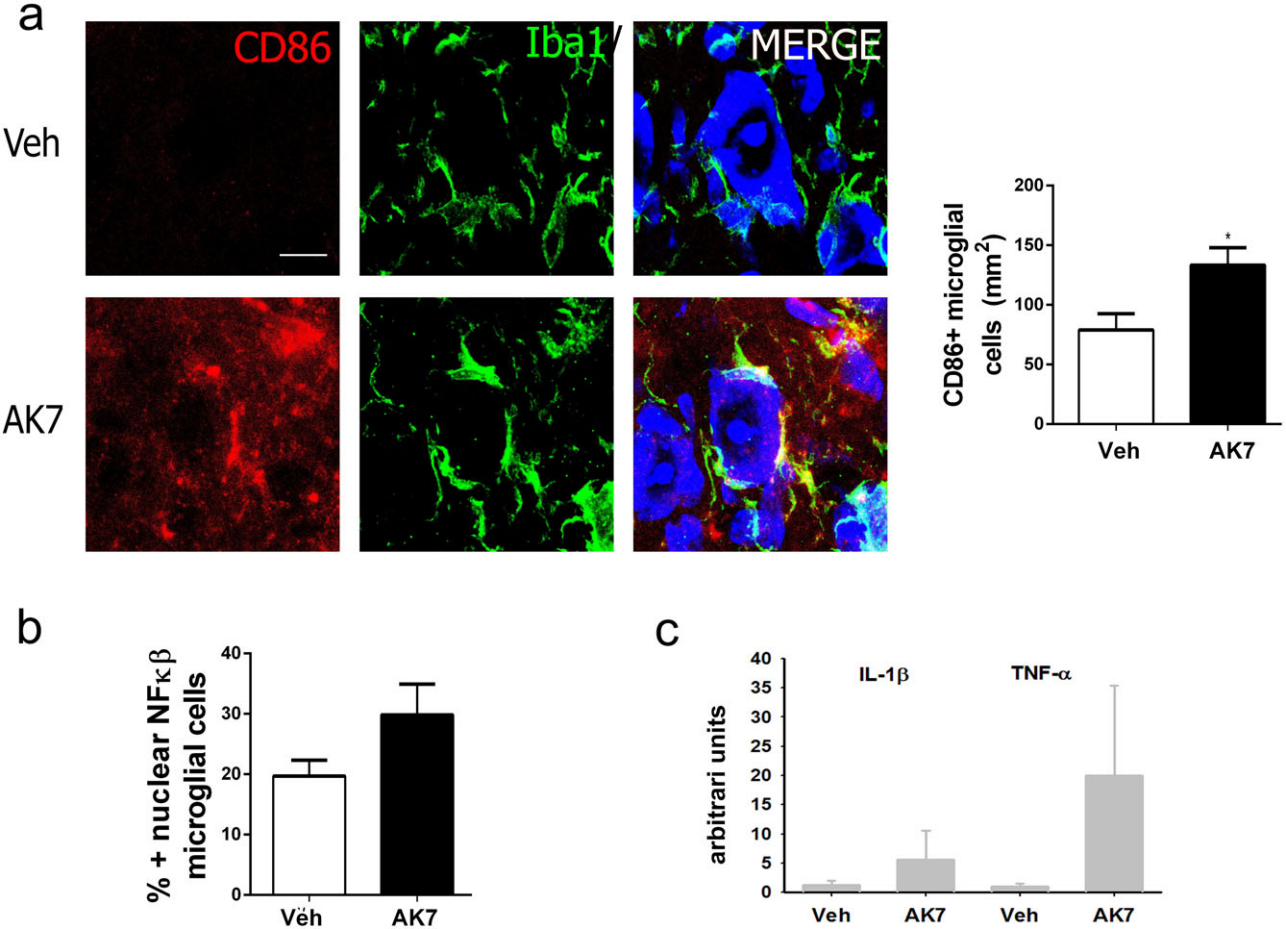
SIRT2 inhibition exacerbates microglial reaction and compromises MN survival after HA. **a** (Left) Microphotographs of samples stained with CD86 (red), a marker of the M1 phenotype, and Iba (green), a microglia marker, and counterstained with DAPI and Fluoro-Nissl (blues) from vehicle or AK7-treated HA-injured animals at 3 dpi. Scale bar = 10 μm. (Right) Histogram showing the means ± SEMs of the percentages of microglial cells with positive CD86 labeling (*n* = 3 for Veh and *n* = 4 for AK7, *t* test, *p < 0.05 vs. Veh). **b** Histogram showing the means ± SEM of the percentages of microglial cells with positive nuclear NF-κB labeling (*n* = 3 for Veh and *n* = 4 for AK7, *t* test, *p < 0.05 vs. Veh). **c** Histogram of mean values obtained by quantitative real-time PCR for *IL-1β* and *TNFα* mRNAs in the hypoglossal nuclei from Veh or AK7-treated animals at 3 dpi

To confirm that glial cells are critical to MN survival in vivo, we analyzed AK7-treated injured animals, and we observed an increase in CD86, a marker of the M1 phenotype, in microglial cells treated with AK7 at 3 dpi (Fig. 6a). We observed significantly more NF-κB in the nucleus of microglia of AK7-treated injured animals than in controls (Fig. 6b). In addition, we observed an increased in the expression of *IL-1β* and *TNFα* genes upon AK7 treatment (Fig. 6c). Thus, although SIRT2 inhibition might be beneficial for the neuronal survival itself, effects on glial cells must also be considered, since the overproduction of pro-inflammatory cytokines by glia upon AK7 treatment might override all benefits to the neurons.

## Discussion

Communication between nerve cells or nerve cells and their target tissue is dysfunctional in neurodegeneration. In addition to their particular protein hallmarks, all neurodegenerative diseases are characterized by neuronal cell death related to soma isolation due to neurite retraction. Deciphering the programs activated by neurons to cope with this damage may guide development of neuroprotective therapies. The purpose of the present study was to assess the roles of SIRT1 and SIRT2 in neuroprotection of disconnected central neurons using the hypoglossal nerve axotomy model. Axotomized cranial MNs suffered non-apoptotic cell death characterized by the activation of the IRE1α -mediated branch of the UPR that is triggered during ER stress. We demonstrated that pharmacological activation of SIRT1 with NH or overexpression of SIRT1 is beneficial, whereas pharmacological or genetic inhibition of SIRT2 is detrimental for axotomized neuron survival in vivo. Interestingly, in the face of ER stress, SIRT2 inhibition with AK7 exerted neuroprotection in a motoneuron (MN)-like cell line but not in MNs in organotypic culture. The difference in these two systems is the presence of glial cells in the latter. AK7 treatment in the organotypic culture and in the in vivo model promoted overproduction of pro-inflammatory cytokines by microglia that can explain its deleterious effect. In this regard, neuroinflammation disrupted UPR activation under proteasome stress situation^28^.

We demonstrated that activation of SIRT1 with NH is important for neuroprotection of disconnected cranial MNs in mice as it was for root avulsed spinal MNs in rats^5^. We recently reported that treatment with NH produces long-lasting neuroprotection and also accelerates nerve regeneration and reduces muscle atrophy in a pre-clinical model of RA^4^. We previously showed that SIRT1 activation was necessary for NH neuroprotection, although this drug composition targets multiple proteins^5^. SIRT1 has been shown to be important for neuroprotection in several models of neurodegenerative diseases (e.g., Alzheimer’s disease, Huntington’s disease)^29–31^ and is crucial in cerebral ischemia and preconditioning^32^. We demonstrated herein that either NH-induced activation or overexpression of SIRT1 boost endogenous mechanisms of neuroprotection after injury, as it is the ATF6-GRP78 branch of UPR^5^. Further future investigations would be worth doing to unravel the mechanisms underlying this activation done by SIRT1. Nevertheless, the data shown here confirm that activation of SIRT1, in particular through the treatment with NH, is a promising therapeutic option for disconnected neurons.

We expected that AK7 and deletion of SIRT2 would be neuroprotective since SIRT2 inhibition is reportedly beneficial in some age-related disorders such as Parkinson’s disease^12, 33–36^ and Huntington disease models^37, 38^. In contrast, our results indicated that AK7 treatment was deleterious for disconnected MN survival in the mouse model. Although in cell lines the treatment was beneficial, in the SCOC model, which more accurately reflects the complex microenvironment of the nervous system, AK7 did not enhance MN survival. In our organotypic and in vivo models, AK7 treatment induced microglia cells to generate pro-inflammatory cytokines, which might override the treatment-induced protection of neurons. After axotomy, the primary injury induces inflammatory responses with cytokines and chemokines released into the peri-lesional areas. Microglial activation, which propagates inflammation into neighboring tissue, may lead to impaired neuronal survival in the peri-lesional regions as a result of the generation of cytokines and reactive oxygen species. Our data indicate that AK7 treatment results in enhanced microglial activation at the injury site, and this suggests that SIRT2 inhibition might aggravate the acute inflammatory response. SIRT2 interacts with p65, an NF-κB subunit, in the cytoplasm and deacetylates lysine 310 in vitro and in vivo^39^. After TNFα stimulation, p65 is hyper-acetylated in SIRT2-deficient cells, which results in increased expression of a subset of p65-target genes^39^. This is in agreement with recent reports that indicate that SIRT2 acetylates p65 to influence pro-inflammatory gene transcription in microglia^40, 26, 8^. Together, these data indicate that post-translational deacetylation of p65 by SIRT2 might be a mechanism that contributes to its anti-inflammatory properties.

Our results are in agreement with other reports that AK7 is not beneficial under conditions where the control of the microglial response is crucial for neuronal survival such as in amyotrophic lateral sclerosis^12^ and traumatic brain injury^41^. Another study using *SIRT2* knockout mice revealed an increase in microglial activation and pro-inflammatory cytokines upon intracortical injection of lipopolysaccharide^27^. *SIRT2* deletion also promotes inflammatory responses in a dextran sulfate sodium-induced model of colitis^42^. When recombinant SIRT2 protein is transduced into murine macrophages, it inhibits lipopolysaccharide-induced expression of cytokines as well as activation of NF-κB and MAPKs^43^, further demonstrating a role for SIRT2 activation in the suppression of the inflammatory response. However, other contradictory results imply that SIRT2 might play a detrimental role under inflammatory conditions. In one study, SIRT2 inhibition by genetic and pharmacological mediators resulted in reactive oxygen species production by macrophages via a mechanism involving degradation of IκBα and nuclear translocation of p65^44, 45^. A very interesting recent study demonstrated that release of cytokines such as *IL-1β* and *TNFα* by classically activated neuroinflammatory microglia induce a subtype of astrocytes termed A1-reactive astrocytes that are neurotoxic for axotomized neurons; these astrocytes are present in patients with human neurodegenerative diseases^46^. Finally, in a totally different way but in agreement to the detrimental effects of the SIRT2 loss-of-function is a recent study reporting axonal degeneration observed in the aged SIRT2-KO mice^47^.

Overall, the differences in the systems studied might explain discrepant results. Our data indicate that caution must be used when extrapolating results obtained in cell culture systems to therapy design.

## Materials and methods

### Animals and surgical procedures

The *SIRT2*^−/−^ C57-BL6 mice were originally generated by Dr. Qiang Tong at The Human Genetics Institute of New Jersey (Piscataway Township, NJ, USA)^25^. Mice with an extra copy of murine form of SIRT1 gene (*tg-SIRT1*) were a kind gift from Dr. Jesús Ruberte (CBATEG, Universitat Autònoma de Barcelona, Barcelona, Spain). This transgenic line was generated by Manuel Serrano at the Tumor Suppression Group, Spanish National Cancer Research Center in Madrid (Spain) following the protocol described in Ref. ^24^.

WT female C57BL/6 (Charles River Laboratories, Wilmington, MA, USA) and KO-SIRT2 mice aged 2 months, and tg-SIRT1 mice weighing an average of 24.92 ± 1.66 g (Animal Service, Universitat Autònoma de Barcelona), were maintained under standard conditions of temperature and light and fed with food and water ad libitum. Surgical procedures were performed under anesthesia with ketamine (90 mg/kg, intramuscularly (i.m.)) and xylazine (10 mg/kg, i.m.). We carried out axotomy of the hypoglossal nerve as described elsewhere^17^. Briefly, the right digastric muscle was opened using blunt-end dissection with a pair of scissors, and the right hypoglossal nerve was exposed. We transected the nerve with a pair of scissors at the proximal side of the hypoglossal nerve bifurcation and removed 3 mm from the distal stump. Finally, we separated the nerve stumps to avoid spontaneous axon regrowth. The muscle was sutured, and the wound closed by planes and disinfected with povidone iodine. The animals were allowed to recover in a warm environment. NH is composed of acamprosate (Merck, Darmstadt, Germany) and ribavirin (Normon, Madrid, Spain)^5^ For in vivo experiments mice were given these compounds in drinking water replaced every 3 days. The concentration of acamprosate was 2.2 mM and that of ribavirin was 1 mM. AK7 (Sigma-Aldrich, Saint Louis, MO, USA) was dissolved in Dimethyl sulfoxide (DMSO), and the stock solution was dissolved in saline. It was administered at 20 mg/kg i.p. daily. All procedures involving animals were approved by the ethics committee of Universitat Autònoma de Barcelona and followed the European Council Directive 2010/63/EU.

### In vitro models

NSC-34 cells were cultured in Dulbecco’s modified Eagle’s medium high-glucose (DMEM, Biochrom, Berlin) supplemented with 10% fetal bovine serum and 1× penicillin/streptomycin solution (Sigma-Aldrich) on collagen-coated plates (Thermo Fisher, Waltham, MA, USA) in a humidified incubator at 37 °C under 5% CO_2_. We transfected 1 × 10^6^ cells with 2 µg plasmid encoding GFP or SIRT1 under the control of a cytomegalovirus promoter using the Amaxa Nucleofector II (Lonza, Norwalk, CT, USA) and the Nucleofactor V kit (Lonza) following the manufacturer’s recommendations. After 4 days of cell culture without changing the medium, cells were differentiated, and freshly prepared drugs diluted in DMEM were added. For ER stress we added 1 μg/ml TUN (TUN, Sigma-Aldrich). Treatments with NH (55 μM acamprosate and 1 μM ribavirin (Sigma-Aldrich, Saint Louis, MO, USA) or 25 μM AK7 were performed concomitantly with TUN treatment. After 24 h, we analyzed cell viability by incubating the cells with 5 mg/ml 3-(4,5-dimethylthiazol-2-yl)-2,5-diphenyltetrazolium bromide (MTT) solution for 4 h at 37 °C and, after medium removal, the MTT salts were dissolved in DMSO. After 5 min, absorbance at 570 nm was measured with a Biotek Elx800 microplate reader (Winooski, VT, USA). The percentage of surviving cells was obtained by comparing each group with the absorbance value derived from control on each plate.

We prepared SCOCs from lumbar sections of 8-day-old Sprague–Dawley pups as previously described^26^. Briefly, we collected the spinal cords from the pups and placed them into cold high-glucose-containing (6.4 mg/ml) Gey’s balanced salt solution (Sigma-Aldrich, Steinheim, Germany). After removing the meninges and roots, we cut spinal cords into 350-μm transverse sections with a McIlwain Tissue Chopper. Four lumbar sections were transferred onto 30-mm diameter, 0.4-μm Millicell-CM nets (Millipore, Billerica, MA, USA) in 6-well plates (Thermo Fisher Scientific) containing 1 ml of medium (50% (v/v) minimal essential medium, 2 mM glutamine, 25 (v/v) Hank’s balanced solution (Sigma-Aldrich) supplemented with 25.6 mg/ml glucose and 25 mM HEPES, pH 7.2). Cultures were maintained at 37 °C in a 5% CO_2_ humidified environment. The medium was unchanged during the first week of culture and was then changed twice per week. After 15 days, we added 1 μg/ml TUN alone or in combination with NH or AK7. At 6 h and at 2 days post treatment, we fixed the SCOCs with 4% paraformaldehyde in a 0.1 M phosphate buffer (pH 7.2) for 1 h at room temperature. We washed the SCOCs with 0.3% Triton X-100 in Tris-buffered saline (TBS; 50 mM Tris, pH 7.6, 150 mM NaCl) solution. SCOCs were blocked with 10% normal donkey serum in TBS and incubated 2 days with mouse anti-SMI-32 (1:1000; Biolegend, San Diego, CA, USA) at 4 °C. Then, we washed the SCOCs with 0.1% Tween-20 in TBS solution and incubated with Alexa 488-conjugated donkey anti-mouse antibodies (1:200; Jackson Immunoresearch, West Grove, PA, USA) for 2 h at room temperature. After several washes, we counterstained with 4',6-diamidino-2-phenylindole (DAPI) and mounted with Fluoromount-G mounting medium (SouthernBiotech, Birmingham, AL, USA). Samples were photographed with an Olympus DP76 digital camera (Olympus, Shinjuku, Tokyo, Japan) attached to the microscope (Olympus BX51). MN survival was assessed by counting all SMI-32-positive neurons at the ventral horn for each spinal cord hemisection. Six different SCOCs were used for each experimental condition.

### Immunohistochemistry and image analysis

After deep anesthesia with pentobarbital, we transcardially perfused the animals with a saline solution containing 10 U/ml heparin, followed by 4% paraformaldehyde in a 0.1 M phosphate buffer, pH 7.2, for tissue fixation at 3, 7, or 21 dpi (*n* = 4 for each condition). Brainstems were removed and post-fixed in the same buffer overnight at 4 °C hours and cryopreserved in 30% sucrose until processed. We isolated the brainstem zone containing the hypoglossal nuclei using a brain mold, and we cut it into serial transversal sections (10 µm thick) with the aid of a cryotome (Leica, Heidelberg, Germany) on gelatinized slides and preserved them at −20 °C until use. For neonatal rats, we perfused them as explained previously at 10 dpi, extracted the L4–L6 spinal cord segment, and cut it into transversal serial sections of 20 µm thick. We treated the brainstem spinal cord or SCOC slides with TBS with 0.3% Triton X-100 and 10% bovine serum for 1 h and incubated them with the following primary antibodies: rabbit anti-Iba1 (1:1000, Wako, Osaka, Japan), rabbit anti-SIRT1 (1:100, Millipore), rabbit anti-Ac-H3K9 (1:50, Millipore), rabbit anti-Ac-p53K373 (1:500, Millipore), rabbit anti-SIRT2 (1:200, Sigma-Aldrich), mouse anti-α-tubulin (1:500; Sigma-Aldrich), mouse anti-acetylated α-tubulin (1:500; Hybridoma Bank, Iowa City, IA, USA), mouse anti-anti-SMI-32 (1:1000; Biolegend), rabbit anti-p65 (1:1000, Cell Signaling, Danver, MA, USA), rat-anti-CD86 (1:200; BD Biosciences, San Jose, CA, USA), rabbit anti-Casp3.Act (1:200; Cell Signaling), and rabbit anti-AIF1 (1:200; Antibodies-Online, Atlanta, GA, USA). After several washes with 0.1% Tween-20 in TBS solution, the sections were incubated for 2 h with donkey anti-rabbit or anti-mouse antibodies (1:200; Jackson Immunoresearch) conjugated to Cy-3, Cy-2, Alexa 488, or Alexa 594 and washed with 0.3% Triton X-100 in TBS. We counterstained the sections with DAPI (Sigma-Aldrich) or NeuroTrace Fluorescent Nissl Stain (Molecular Probes, Leiden, Netherlands) and mounted sections with Fluoromount-G mounting medium (SouthernBiotech). Sections to be compared were processed together on the same slide and on the same day. Images from sections of different treatments and controls were taken under the same exposure time, sensitivity, and resolution for each analyzed marker.). Confocal images were obtained using two separate photomultiplier channels with a 1.4 numerical aperture oil-immersion objective of ×20 or ×40. Images were separately projected and merged using a pseudocolor display. We analyzed fluorescence signal intensity using the ImageJ software (National Institutes of Health, Bethesda, MD, USA; available at http://rsb.info.nih.gov/ij/). The analysis of the images to be compared was performed the same day. We delimited an area as region of interest (ROI) with the aid of Fluoro-Nissl labeling for MN cytoplasm or DAPI for MN nuclei. The integrated density inside the ROI was obtained for at least 15 MNs/animal per marker from three 100-μm sections from distant regions.

### Fluoro-Jade C staining

Brainstem sections were randomly selected and stained with Fluoro-Jade C (Chemicon, Tokyo, Japan) following the manufacturer’s protocol. In brief, the slices were immersed in a solution of 80% EtOH with 1% of NaOH for 5 min, then rinsed for 2 min in 70% EtOH and 2 min in distilled water, and incubated for 10 min with 0.06% permanganate solution. After washing with water, the slices were transferred to a solution of 0.0001% of Fluoro-Jade C dissolved in 0.1% acetic acid and were incubated for 10 min. Then, the slices were washed with water, counterstained with DAPI, and mounted with dibutylphthalate polystyrene xylene (Sigma). Confocal images were taken within several hours of the staining to avoid fluorescence loss.

### Motor neuron counting

Sixteen brainstem sections separated by at least 50 μm and including the hypoglossal nuclei were randomly selected and stained with Fluorescent Neurotracer (Life Technologies, Carlsbad, CA, USA) following the manufacturer’s protocol. Sequential microphotographs from the injured and contralateral MN hypoglossal were taken using an Olympus DP76 digital camera attached to the microscope (Olympus BX51). The MNs with diameters >17 μm (area of 320 μm_2_)^48, 49^, prominent nucleous, polygonal shape, and located in the hypoglossal nucleus were counted. For comparisons, the percentage of MN survival was calculated by comparison to numbers at the injured site with numbers at the contralateral site for each section.

### Western blot

Control and HA-injured mice with or without NH treatment at 3 dpi (*n* = 4 per group) were deeply anesthetized with pentobarbital, and whole brain tissue was obtained for western blot analysis. Samples were snap frozen in liquid nitrogen. Hypoglossal nuclei were removed and homogenized in lysis buffer (20 mM HEPES, pH 7.2, 250 mM sucrose, 1 mM EDTA, 1 mM EGTA, and a cocktail of protease (Sigma) and phosphatase inhibitors (Roche, Manheim, Germany)) using a Potter homogenizer on ice. The lysate was centrifuged for 20 min at 800 × *g* at 4 °C, and the supernatant (the cytosolic fraction) was collected. The pellet was mixed with nuclear buffer (20 mM HEPES, pH 7.2, 0.1% Triton X-100, 0.2 mM EGTA, 0.2 mM EDTA, 1 M KCl, and a cocktail of protease (Sigma) and phosphatase inhibitors (Roche)) by vortexing at 1400 rpm for 20 min at 4 °C. After centrifuging at 10,000 × *g* for 10 min at 4 °C, the supernatant was collected (the nuclear fraction). Finally, we quantified the amount of protein in cytosolic and nuclear fractions using the BCA assay (Pierce Chemical Co., Rockford, IL, USA). For western blotting, we mixed equal amounts of samples from four animals and loaded 10 μg of cytosolic or nuclear fractions onto a 10% sodium dodecyl sulfate-polyacrylamide gel. After electrophoretic separation of proteins and transfer to a PVDF membrane in a Bio-Rad Cubette System (Bio-Rad Laboratories, Hercules, CA, USA) in 25 mM Tris, pH 8.4, 192 mM glycine, and 20% (v/v) methanol. After blocking with TBS, 0.1% Tween-20, 5% milk, the membrane was incubated overnight at 4 °C with the primary antibody: rabbit anti-IRE1α (1:1000), rabbit anti-XBP1 (1:1000), rabbit anti-phosphor-PERK (pPERK,1:1000), rabbit anti-PERK (1:1000) (Cell Signaling, Denvers, MA, USA), rabbit anti-ATF6 (1:1000, Santa Cruz Biotech, Santa Cruz, CA, USA), or GRP78 (1:1000, Stressgen Biotechnologies, Victoria, BC, Canada). The following day, membranes were washed and then incubated for 1 h with an appropriate secondary antibody conjugated with horseradish peroxidase (1:5000, Vector Laboratories, Burlingame, CA, USA) at room temperature. Proteins were developed using a chemiluminescence method (ECL Clarity Kit, Bio-Rad Laboratories), and the images were captured and analyzed with Image Lab Software (Bio-Rad Laboratories).

### RNA extraction, reverse transcription, and real-time PCR

For PCR analysis, total RNA was extracted using the Tripure Isolation Reagent (Roche) according to the instructions of the manufacturer. Reverse transcription was performed using random hexamers primers exactly as previously described^50^. PCR was performed in an ABI Prism 7000 sequence detector (Applied Biosystems, Madrid, Spain) using cDNA diluted in sterile water as template. *TNFα*, *IL-1β*, *Grp78*, and the control housekeeping genes were amplified using specific Taqman probes supplied by Applied Biosystems. Some primers were used to amplify *Chop* (forward (F): 5′-CCCCAGGAAACGAAGAGGAAGAATC-3′; reverse (R): 5′-CTACCCTCAGTCCCCTCCTCAGCAT-3′), *Xbp1total* (F: 5′-GCTTGTGATTGAGAACCAGG-3′; R: 5′-GAGGCTTGGTGTATACATGG-3′), and *Xbp1s* (mix of F: 5′-GCTTGTGATTGAGAACCAGG-3′; R: 5′-GGCCTGCACCTGCTGCGGACTC-3′ and F: 5′-GAGTCCGCAGCAGGT-3′; R: 5′- GAGGCTTGGTGTATACATGG-3′). Threshold cycle (Ct) values were calculated using the software supplied by Applied Biosystems.

### Statistical analyses

All data are presented as means ± standard errors of the means. The statistical analysis was performed with GraphPad Prism 5 software (San Diego, CA, USA) using unpaired *t* test (one or two tailed) or one-way analysis of variance followed by Bonferroni’s multiple test. Differences were considered significant at *p* < 0.05.

## Electronic supplementary material

Figure S1

Figure S2

Supplementary figure legends
