# Supplementary figures and images for: SIRT1 activation with neuroheal is neuroprotective but SIRT2 inhibition with AK7 is detrimental for disconnected motoneurons
Source: Cell Death Dis. 2018 May 10;9(5):531. doi: 10.1038/s41419-018-0553-6 (PMC5945655; doi:10.1038/s41419-018-0553-6)

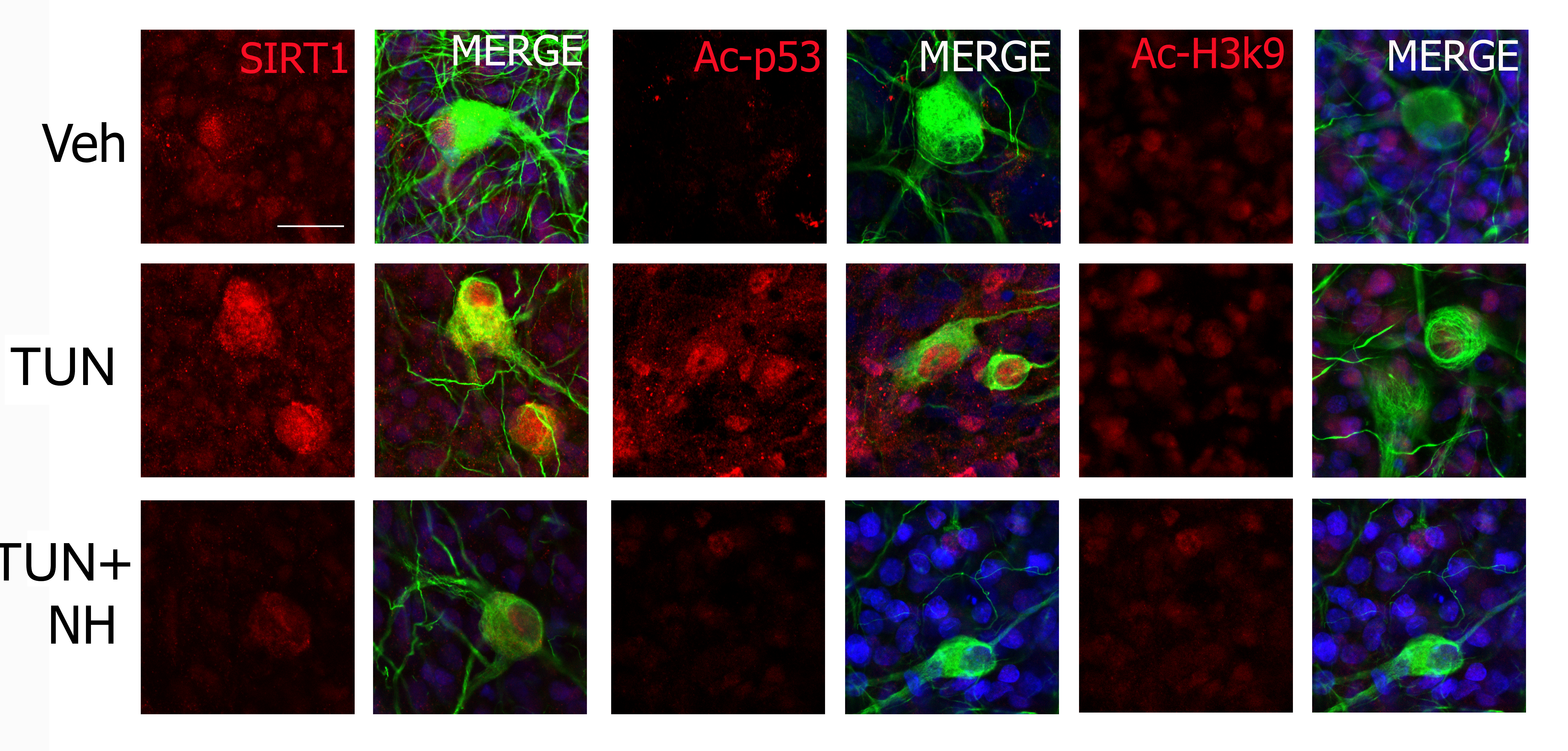

Supplement: Supplementary file 1 — Figure S1 [file 41419_2018_553_MOESM1_ESM.jpg]

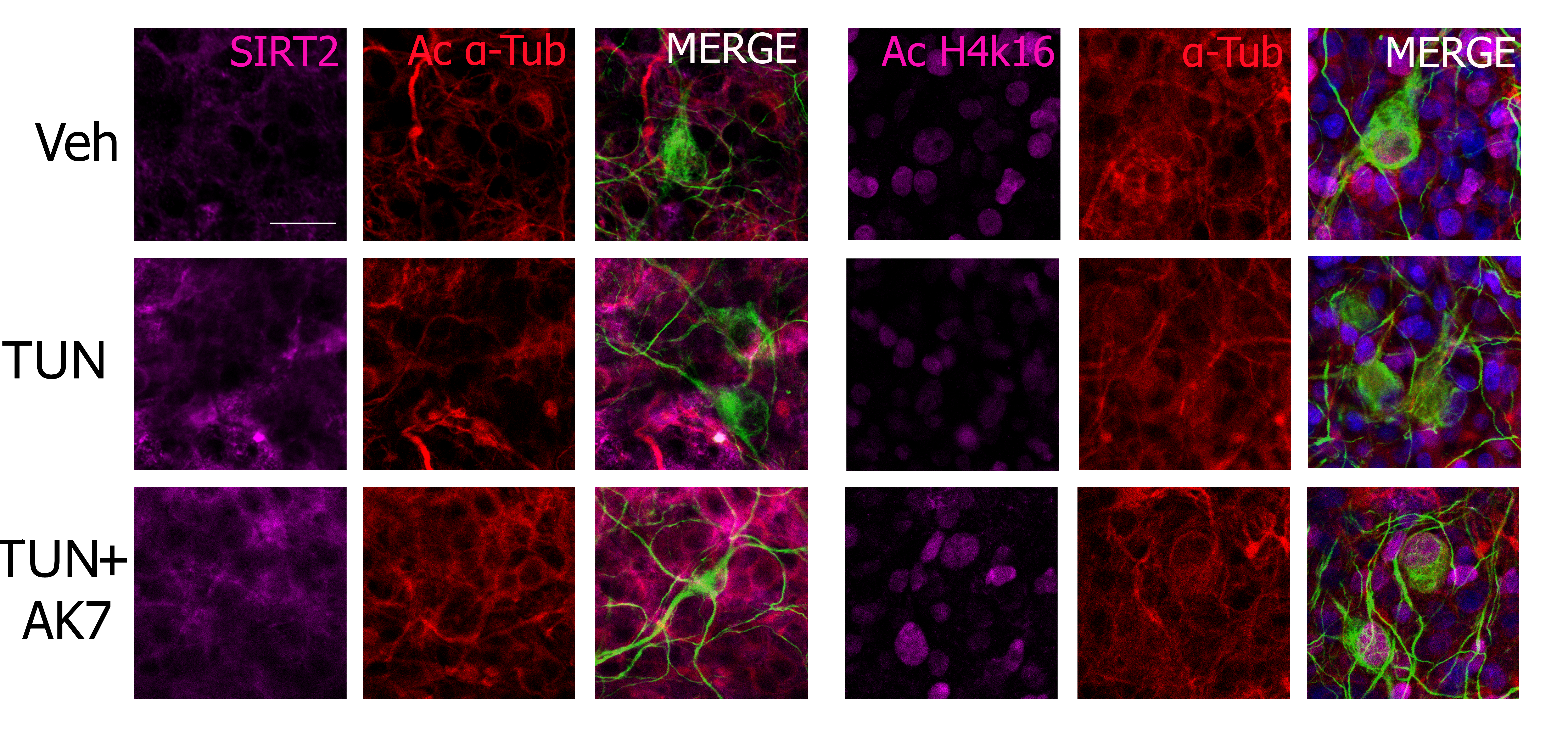

Supplement: Supplementary file 2 — Figure S2 [file 41419_2018_553_MOESM2_ESM.jpg]
