# Supplementary material for: SIRT1 activation with neuroheal is neuroprotective but SIRT2 inhibition with AK7 is detrimental for disconnected motoneurons
Source: Cell Death Dis. 2018 May 10;9(5):531. doi: 10.1038/s41419-018-0553-6 (PMC5945655; doi:10.1038/s41419-018-0553-6)
Supplement: Supplementary file 3 — Supplementary figure legends [file 41419_2018_553_MOESM3_ESM.docx]

**Figure S1. TUN reduces SIRT1 activity and NH reverts this blockage.** (**a**) Confocal microphotographs of SCOCs immunolabeled for SIRT1, Ac-p53K373, and Ac-H3K9 in red, SMI-32 in green, and DAPI in blue after 2 days with indicated treatments. Scale bar = 25 µm.

**Figure S2. ER stress increases SIRT2 activity and AK7 blocks SIRT2 activity.** (**a**) Confocal microphotographs of SCOCs immunolabeled for SIRT2 or Ac-H4K16 in magenta, acetylated or total α-tubulin in red, SMI-32 in green, and DAPI in blue 6 h after Veh, TUN, or TUN plus AK7 treatment. Scale bar = 25 µm.
